# Airway management of ruptured pulmonary artery “Rasmussen” aneurysm and massive hemoptysis

**DOI:** 10.1186/s13104-015-1313-7

**Published:** 2015-08-12

**Authors:** Madiha Syed, Jill Irby

**Affiliations:** University of Arkansas for Medical Sciences, 4301 West Markham Street, Slot 515, Little Rock, AR 72205 USA

**Keywords:** Tuberculosis, Massive hemoptysis, Airway management

## Abstract

**Background:**

Pulmonary tuberculosis is caused by *Mycobacterium tuberculosis* and its manifestations may include parenchymal, airway, vascular, pleural, mediastinal and chest wall lesions. Hemoptysis is a common complication of the disease. Massive hemoptysis occurs in about 8 % of cases; with associated mortality ranging from 5 to 25 % Massive hemoptysis secondary to pulmonary artery aneurysm rupture is a rare phenomenon presenting unique challenges in airway management and stabilization of oxygenation, ventilation and blood pressure.

**Case history:**

We present a case of a patient with necrotizing pulmonary tuberculosis complicated by a ruptured pulmonary artery “Rasmussen” aneurysm requiring emergent intubation and embolization.

**Conclusion:**

Massive hemoptysis should be treated as a medical emergency requiring the coordinated care of multiple specialists including intensivists, interventional radiologists, anesthesiologists, and surgeons. Airway management and stabilization of cardiorespiratory status should be the highest priority, followed by timely diagnostic procedures to localize the site and cause of the bleeding.

**Electronic supplementary material:**

The online version of this article (doi:10.1186/s13104-015-1313-7) contains supplementary material, which is available to authorized users.

## Background

Pulmonary tuberculosis is caused by *Mycobacterium tuberculosis* and its manifestations may include parenchymal, airway, vascular, pleural, mediastinal and chest wall lesions. Hemoptysis is a common complication of the disease. Massive hemoptysis occurs in about 8 % of cases, with associated mortality ranging from 5 to 25 % [1]. Most of the cases of hemoptysis originate from hypertrophied bronchial arteries; a few cases arise from pulmonary artery aneurysms which arise secondary to focal weakening of the artery wall from inflammatory infiltrates [2, 3].

## Case report

A 35 year African American female presented to the emergency room (ER) with history hemoptysis, fever and dyspnea. She had no significant past medical history. Her past surgical history was significant for hysterectomy, tubal ligation and ankle surgery. Social history was significant for smoking 1.5 packs/day and occasional marijuana use. On clinical exam she was febrile (102 °F), tachycardic (heart rate 122 beats/min), blood pressure 108/71, respiratory rate 22 per min and SPO_2_ 100 % on room air. Laboratory evaluation was significant for anemia (Hb 7.8 g/dL; Hct 26.8 %), hyponatremia with Na of 125 meq/L, and metabolic acidosis with bicarbonate of 19 meq/L. Chest X-ray showed bilateral upper lobe necrotizing pneumonia and a left lower lobe infiltrate.

She was evaluated with flexible bronchoscopy, which revealed blood trickling from the left main stem, and bronchoalveolar lavage which was positive for acid fast bacilli. She was started on rifampin, isoniazid, pyrazinamide, and ethambutol. CT angiogram was negative for any bleeding and showed bilateral cavitary lesions (the largest lesions were on the left, measuring 6.6 × 6.4 cm).

On hospital day 3, the patient developed massive hemoptysis. Her hemoglobin dropped from 8 to 6.8 g/dL and she lost greater than 500 mL of blood within 5–10 min. Vital signs at that time were: temperature 99.1 °F, heart rate 110–120 beats/min, respiratory rate 30–40 breaths/min, blood pressure 90–98/57–66 mmHg and SpO_2_ 97 % on room air. Primary medical team contacted the anesthesia team for emergent intubation in the step-down unit. In light of the massive hemoptysis and challenges of securing an airway obscured by blood, the decision was made to take patient to the operating room for resuscitation and intubation. In the operating room the patient was pre-oxygenated with 100 % FiO_2_ in the sitting position and a rapid sequence induction with cricoid pressure was performed with etomidate 20 mg IV and succinylcholine 100 mg IV. A videolaryngoscope was used to attempt to place a 35 French left side double lumen tube. The glottis view was initially obscured by fresh blood. After suctioning, a Grade I view was obtained. An attempt was made to place a 35 French left sided double lumen tube, this was not successful due to fresh blood obscuring the view and resistance to passage of the double lumen tube. This was immediately followed by direct laryngoscopy and placement of a 7.5 single lumen endotracheal tube (ETT). A large amount of blood was noted in the oropharynx and ETT tube during and after intubation. Blood was also noted in expiratory circuit and end tidal CO_2_ line and these were exchanged for fresh circuits. The patient became severely bradycardic after intubation (HR 30–40 beats/min) requiring atropine 0.4 mg and epinephrine 0.5 mg. Two 14 gauge intravenous lines were placed in right and left external jugular veins and connected to high flow fluid infusers. A radial arterial line was placed. The patient was transfused with six units of packed red cells, two units of fresh frozen plasma and two units of platelets. During resuscitation, the patient was manually ventilated at a rate of 30 breaths/min, with the airway pressure release valve closed. Tidal volumes of only 150–200 mL were achieved. After stabilization of vital signs, the single lumen tube was exchanged for a 35 French left double lumen tube with a 14 French Cook Airway Exchange catheter and patient was transported to interventional radiology suite for embolization.

Prior to the start of the embolization, we attempted to ascertain position of double lumen tube with a flexible bronchoscope but were unable to visualize landmarks secondary to hemoptysis. Fluoroscopy was utilized to position tube and an attempt was made to isolate the left lung. The patient did not tolerate lung deflation with tidal volumes deceasing to 70 mL and absence of breath sounds bilaterally. The interventional radiologist identified three pseudoaneurysms in the left upper lobe of which two had ruptured. All three were embolized with NBCA (*N*-butyly-2-cyanoacrylate) glue. She received 2 more liters of normal saline and intermittent boluses of phenylephrine during the procedure. After the procedure, the double lumen tube was exchanged for a single lumen tube with a Cook Airway exchange catheter; she was sedated with midazolam 2 mg and transported to MICU.

The patient required ventilator support for 4 days after which she was extubated and given oxygen via a 50 % Venti mask. Her post-extubation hospital course was complicated by delirium which improved and she was discharged to home with oxygen on hospital day 15. Arterial blood gases during the procedure and hospital admission are in Table 1.

**Table 1:** Arterial blood gases during procedure and hospital admission

Location	Day	Time	FiO_2_	pH	PCO_2_	PO_2_	HCO_3_	BE	Hb/Hct	SpO_2_	Lactate
OR	1	0133	1.0	6.94	88	117	18.9	−13.6	9.4/28	96.3	1.9
OR	1	0302	1.0	7.11	63	298	20	−9.7	10.6/32	96.9	0.6
ICU	1	0523	1.0	7.13	54	89	18	−11.4	13.6/41	93.9	–
ICU	1	0731	0.6	7.22	44	65	18	−9.4	11.6/35	91.1	–
ICU	1	2100	1.0	7.35	36	230	19.9	−5.2	8.8/26	96.6	–
ICU	4	0315	0.4	7.45	38	96	26.4	2.3	8.4/25		
Floor bed	11		0.21 (room air)	7.48	42	48	31.3	7.1	7.8/23	81.7	–

Written consent was obtained from patient to publish details of the case. Case report was prepared with according to the guidelines in the CARE checklist (Additional File 1).

## Discussion

The most common manifestations of tuberculosis tend to be pulmonary although virtually any organ system may be affected. Our patient presented with a combination of necrotizing pneumonia, bilateral cavitary lesions and Rasmussen pseudoaneurysms. Vascular complications may involve both the arterial and venous circulation. Bronchial arteries may become hypertrophied especially in areas of bronchiectasis, the mediastinum, and central airway and may appear as nodular or tubular structures on high resolution CT. Care must be exercised by bronchoscopist to avoid biopsying these lesions [4].

Rasmussen aneurysms are rare phenomena. These aneurysms arise from segmental pulmonary arteries, and occur secondary to infiltration of granulation tissue into the adventia and media. This is replaced in time by fibrin leading to thinning of the vessel wall and subsequent aneurysm formation and rupture leading to hemoptysis [5]. A study reviewing autopsy findings in patients with chronic cavitary TB showed an incidence of 5 % [5]. Another retrospective series of 189 patients utilizing multidetector CT reported a prevalence of 6.9 % in patients with massive hemoptysis and TB [3].

First line investigation in patients with massive hemoptysis requiring intensive care is fiberoptic bronchoscopy followed by urgent bronchial artery embolization. In patients where embolization fails emergent surgery is the second line option [6]. Postoperative complications may occur in 50 % of cases and fatality rate may be high as 20 %, especially in cases where surgery is performed 24 h after onset of hemoptysis [6].

Massive hemoptysis presents a very challenging scenario in critical care medicine. The first priority is to gain control of airway and ensure oxygenation and ventilation. Intubation with a single lumen tube is recommended [7]. A double lumen tube may be used to get rapid control and isolation of the lungs if the laterality of the bleeding is known. An alternative to a double lumen tube could be placement of a single lumen tube, which can be advanced into the mainstem bronchus on the affected side. The choice of tube should be dictated by the ease with which clots and blood can be removed from the airway as death from hemoptysis occurs not from exsanguination but from asphyxia which is caused by retained blood clots [7]. Following intubation, identifying and treating the cause of hemoptysis should be the next step. Flexible or rigid bronchoscopy to identify the site of the bleeding should be done. Rigid bronchoscopy may be advantageous in removing clots but is not good for visualizing peripheral lesions and requires specialized expertise. Flexible bronchoscopy is easily performed as a bedside procedure. The overall diagnostic accuracy of fiberoptic bronchoscopy in patients with hemoptysis is 10–43 %, and ranges from 0 to 31 % in patients with hemoptysis and normal chest radiographs [1]. In a study by Düpree et al. repeated instillation of cold saline with epinephrine (1:10,000–100,000) by fiberoptic bronchoscopy in intubated patients with massive hemoptysis was successful in 6 out of 7 patients [8]. Recognizing the dual blood supply of the lungs, selective bronchial arteriography should be considered to distinguish bronchial lesions from pulmonary artery lesions like Rasmussen aneurysms. Bronchial artery lesions are responsible for massive hemoptysis in approximately 90 % of cases. CT scans and CXR are also important diagnostic tools and are usually performed prior to bronchoscopy. CT scans may be able to identify the source of hemorrhage in 63–100 % patients with hemoptysis, which is a higher rate than fiber optic bronchoscopy [1]. Fiber optic bronchoscopy and CT scans together provide a better chance of obtaining a diagnosis.

Of note is the alarmingly high mortality rate of 50–100 % for conservative management of massive hemoptysis [9]. Mortality rates for surgery range from 7.1 to 18.2 %, but may increase to 40 % for emergency surgery [10].

## Conclusion

Massive hemoptysis should be treated as a medical emergency requiring the coordinated care of multiple specialists including intensivists, interventional radiologists, anesthesiologists, and surgeons. Airway management and stabilization of cardiorespiratory status should be the highest priority, followed by timely diagnostic procedures to localize the site and cause of the bleeding. Due to the high risk of rebleeding a definitive diagnostic and treatment plan is essential prior to discharging the patient home.

## Consent

Written informed consent was obtained from the patient for publication of this Case report and any accompanying images. A copy of the written consent is available for review by the Editor of this journal.

## Additional file

Additional file 1: Care Checklist of information to include when writing a case report.
